# Complete mitochondrial genome and phylogenetic analysis of *Triplophysa jianchuanensis* (Cypriniformes: Cobitidae)

**DOI:** 10.1080/23802359.2023.2288440

**Published:** 2023-12-07

**Authors:** Yonglan Peng, Yuping Qiu, Guozhu Chen

**Affiliations:** a Yunnan Key Laboratory of Plateau Wetland Conservation, Restoration, and Ecological Services, Southwest Forestry University, Kunming, PR China; b National Plateau Wetlands Research Center/Colloge of Wetlands, Southwest Forestry University, Kunming, PR China; c National Wetland Ecosystem Fixed Research Station of Yunnan Dianchi, Southwest Forestry University, Kunming, PR China

**Keywords:** Mitochondrial genome, *Triplophysa jianchuanensis*, phylogenetic analysis

## Abstract

We used the Illumina MiSeq platform to sequence the complete mitochondrial genome of *Triplophysa jianchuanensis* (Cypriniformes: Cobitidae). The mitochondrial genome of *T. jianchuanensis* is 16,569 bp in length, and its genetic constitution and arrangement are consistent with those of the teleost taxon, containing 13 protein-coding genes, 22 tRNA genes, 2 rRNA genes, an origin of light-strand replication, and a control region. The overall nucleotide base composition was 28.33% A, 27.25% T, 17.77% G, and 26.65% C. Phylogenetic analysis showed that *T. jianchuanensis* was grouped with *T. venusta*. These findings are valuable for further studies on the evolution, genetic diversity, and taxonomy of *Triplophysa*.

## Introduction

*Triplophysa jianchuanensis* (Zheng et al. 2010) belongs to the genus *Triplophysa* within the subfamily Nemacheilinae, and is a recently recorded fish endemic to the Jianhu River drainage of Yunnan Province, China (Zheng et al. 2010). *Triplophysa* is one of the largest genera of these loaches, with nearly 160 species discovered to date (https://researcharchive.calacademy.org/research/ichthyology/catalog/fishcatmain.asp) (Zhao et al. 2022). *Triplophysa* species are found worldwide but mainly inhabit the Tibetan Plateau and its surrounding areas, including karst areas in southwestern China. *T. jianchuanensis* has a narrow distribution in the Erhai Basin, which is a tributary of the middle reaches of the Lancangjiang River, at an altitude of approximately 2000 m. Despite several basic studies on *Triplophysa* species (Wang et al. 2019; Ning et al. 2020; Yang et al. 2020; Wang et al. 2021), the complete mitochondrial genome sequence of *T. jianchuanensis* is not publicly available. Therefore, in this study, we aimed to sequence the complete mitochondrial DNA genome of *T. jianchuanensis* in order to provide basic data pertaining to this species and to better understand its relationship with other Cobitidae species.

## Materials and methods

**Figure 1.**
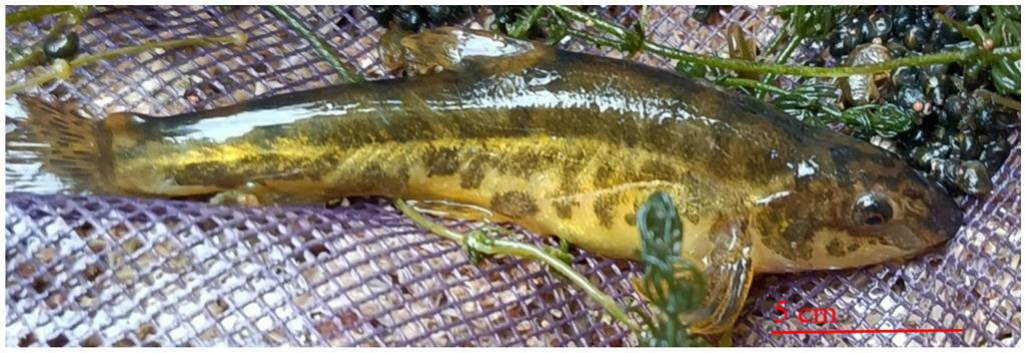
Living specimen of *T. jianchuanensis* (photo by Guozhu Chen). the specimen was collected from the Dali Prefecture, Yunnan Province, China.

The living specimen was collected from Jianhu Lake in Dali Prefecture, Yunnan, China (26°41′N, 99°98′E) in June 2019 (Figure 1). The specimen was preserved at the National Plateau Wetlands Research Center, Southwest Forestry University (http://plateauwetland.swfu.edu.cn/, Guozhu Chen, chenguozhu79@163.com), under voucher number SWFU20190058. Here, the complete mitochondrial genome of *T. jianchuanensis* was sequenced and characterized in detail, which not only enriches the mtDNA data of the genus but also provides a powerful resource for species identification, genetic diversity, and phylogenetic relationships.

**Figure 2.**
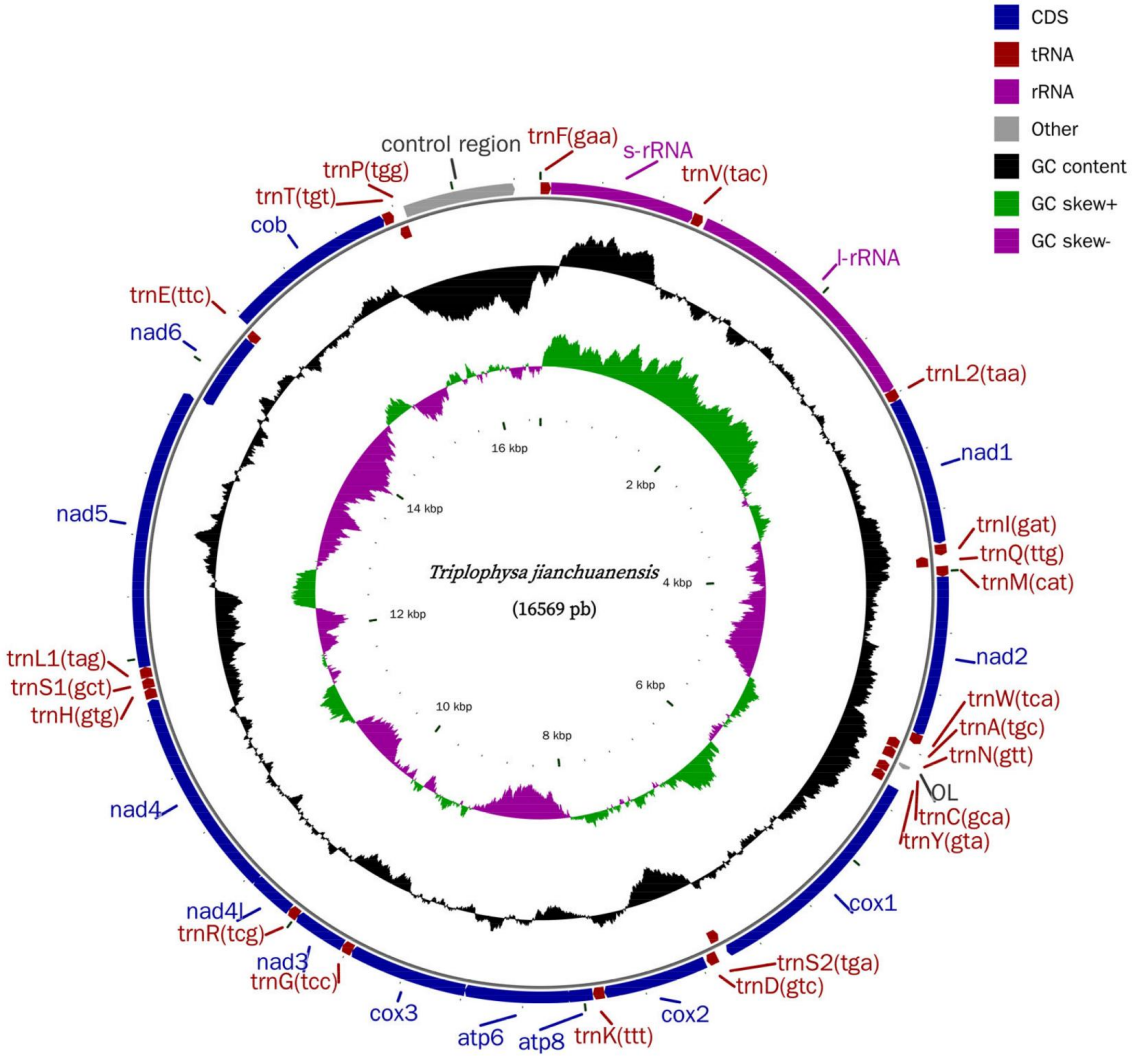
Complete mitochondrial genome map of *T. jianchuanensis* (GenBank: OQ603602). Genes encoded on the light strand are represented on the inner side of the gray circle, while genes encoded on the heavy strand are shown on the outer side.

Total genomic DNA was extracted from the muscle tissue using a tissue genomic DNA extraction kit (Tiangen, Beijing, China). We used the whole-genome shotgun strategy to construct the library, which was then paired-end sequenced using next-generation sequencing on the Illumina MiSeq platform. The depth of coverage is shown in Supplementary Figure S1. The resulting circular consensus sequence was annotated and verified using the MITOS (Bernt et al. 2013) web server (http://mitos2.bioinf.uni-leipzig.de/index.py). Organelle genome maps were drawn using CGView software (Stothard and Wishart 2005) (Figure 2). Sequences used in the phylogenetic analysis were from the NCBI GenBank database; they were aligned with MAFFT (Katoh and Standley 2013). Phylogenetic analysis was performed using the IQ-TREE 2 (Minh et al. 2020).

## Results

### Mitogenome organization

The complete *T. jianchuanensis* mitochondrial genome (accession number: OQ603602) was 16,569 bp, of which 15,802 nucleotides were coding DNA and 767 nucleotides were non-coding DNA, containing 22 tRNA genes, 2 rRNA genes, 13 protein-coding genes, an origin of light-strand replication (O_L_), and a control region (D-loop). The arrangement and composition of the mitochondrial genome were comparable to those of other *Triplophysa* (Wang et al. 2019; Ning et al. 2020; Yang et al. 2020; Wang et al. 2021). The overall base composition was A (28.33%), T (27.25%), G (17.77%), and C (26.65%), as well as an AT bias of 55.58%, which is generally reported for other teleost mitochondrial genomes. Twelve mitochondrial protein-coding genes shared regular ATG initiation, and only cox1 began with GTG, which is similar to other *Triplophysa* fishes (Wang et al. 2019; Ning et al. 2020; Yang et al. 2020; Wang et al. 2021). Four types of termination codons were observed in the protein-coding genes: TAA for nad1, cox1, atp8, atp6, nad41, nad5, and nas6; TAG for nad2 and nad3; T(AA) for cox2, nad4, and cob; and TA(A) for cox3. The total length of the 13 protein-coding genes was 11,426 bp; the longest was nad5 (1839 bp), and the shortest was atp8 (168 bp). The *rrnS* (950 bp) and *rrnL* (1656 bp) genes were located between the *rrnF* and *rrnL2* genes and separated by the *rrnV* gene. The lengths of the 22 tRNA-coding genes ranged from 66 bp to 75 bp. The remaining seven genes were terminated with TAA. All mitogenomic genes were encoded on the H strand, except for *nad6* and 8 tRNA genes (*trnQ, trnA, trnN, trnC, trnY, trnS2, trnE, and trnP*).

### Phylogenetic analysis

**Figure 3.**
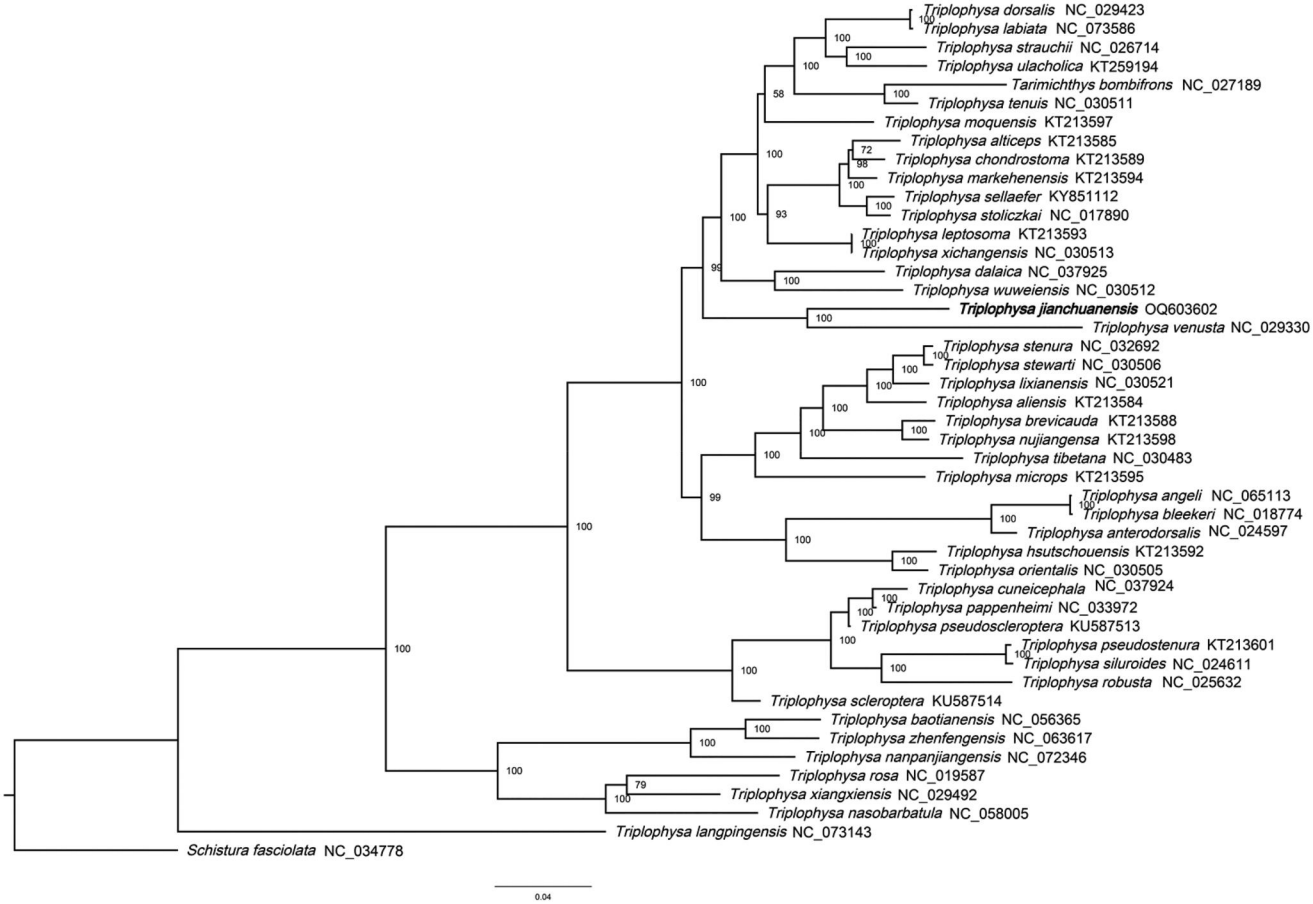
The maximum likelihood phylogenetic tree. The tree was established using complete mitochondrial DNA sequences of 45 *Triplophysa* species and 1 of *Schistura* species; *S. fasciolata* (NC_034746) was set as the outer group. Accession numbers are indicated after the species names. Numbers above or below nodes indicated the bootstrap support values estimated with 1000 replicates.

To assess the phylogenetic relationships of *T. jianchuanensis*, we selected the complete mitochondrial DNA sequences of 45 *Triplophysa* species and 1 *Schistura* species; *Schistura fasciolata* was set as the outer group. The sequences were obtained from GenBank. We used the maximum-likelihood analysis with the best-fitting model GTR + I + G and 1000 bootstrap replicates. In this study (Figure 3), *T. jianchuanensis* and *T. venusta* were found to be sister species with a 100% support value, and both were distributed in Dali Prefecture. *T. jianchuanensis*. had a close relationship with the branches *T. wuweiensis* and *T. dalaica,* with high support values. This newly sequenced complete mitochondrial genome provides valuable information for exploring the genetic diversity and phylogenetic relationships of the *Triplophysa* family.

## Discussion and conclusion

Next-generation sequencing and assembly revealed that the complete mitogenome of *T. jianchuanensis* is 16,569 bp in length. The gene order and composition are identical to those of typical mitogenomes of other teleost fish (Wang et al. 2019; Ning et al. 2020; Yang et al. 2020; Wang et al. 2021). The maximum likelihood tree based on the complete mitochondrial genomes of *T. jianchuanensis* and 47 other species supported the hypothesis that *T. jianchuanensis* constitutes a sister group with *T. venusta*. In conclusion, this study provides important information for future taxonomic, systematic, and genetic studies on *Triplophysa*.

## Supplementary Material

Supplemental Material

## Data Availability

The genome sequence data that support the findings of this study are openly available in the GenBank of NCBI [https://www.ncbi.nlm.nih.gov] under accession no. OQ603602. The associated BioProject, SRA, and BioSample numbers are PRJNA943932, SRR23852298, and SAMN33734297, respectively.
