# Supplementary figures and images for: Complete mitochondrial genome and phylogenetic analysis of Triplophysa jianchuanensis (Cypriniformes: Cobitidae)
Source: Mitochondrial DNA B Resour. 2023 Dec 7;8(12):1330–3. doi: 10.1080/23802359.2023.2288440 (PMC10776075; doi:10.1080/23802359.2023.2288440)

Coverage Map

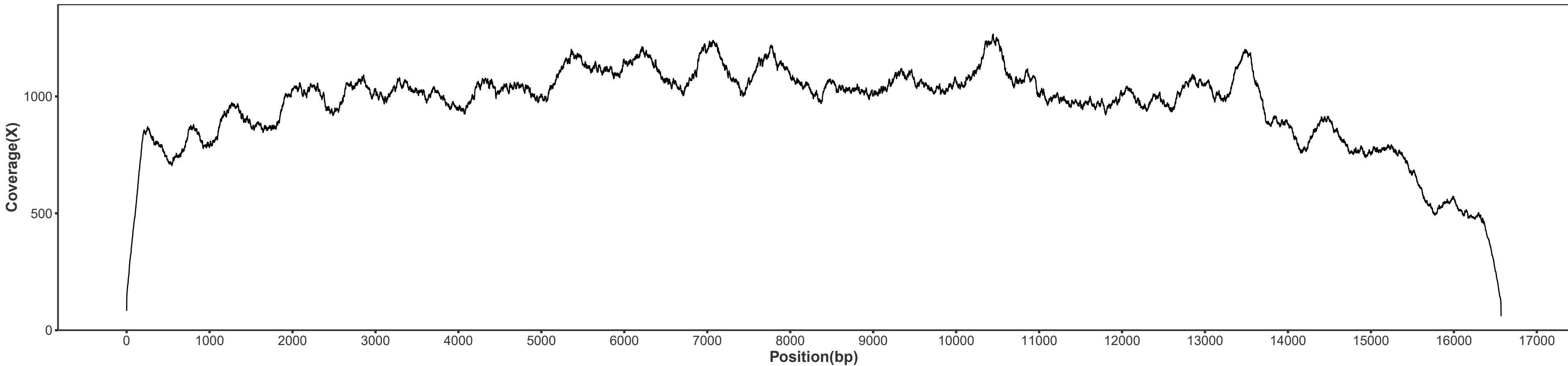

Supplement: Supplemental Material [file TMDN_A_2288440_SM8429.pdf]
